# Descriptive evaluation of student-created infographics: reliability and quality in an undergraduate planetary health and sustainable healthcare online course

**DOI:** 10.3389/fmed.2026.1770689

**Published:** 2026-04-20

**Authors:** Mennatallah Hassan Rizk, Ziad Ahmed Abdel-salam Selim, Hager Abdelaziz Abdallah Ataallah, Soha Rashed, Hanaa Saeed Elhoshy

**Affiliations:** 1 Department of Medical Education, Faculty of Medicine, Alexandria University, Alexandria, Egypt; 2 Faculty of Medicine, Alexandria University, Alexandria, Egypt

**Keywords:** bloom’s taxonomy, descriptive evaluation of students, infographics, inter-rater reliability, sustainable healthcare

## Abstract

**Background:**

Physicians require visual communication skills to convey complex health information effectively. This descriptive evaluation assessed infographic quality and inter-rater reliability as a Bloom’s “create”-level assessment tool within Alexandria University’s third-year Sustainable Healthcare elective, addressing a gap in visual literacy training within the medical curriculum.

**Methods:**

We analyzed 20 collaborative group infographics from the Planetary Health and Sustainable Healthcare online course. Three blinded expert raters independently applied a four-point weighted rubric (total composite score 0–4.0). Reliability was quantified using a two-way mixed-effects intraclass correlation coefficient (ICC) (absolute agreement and average measures) and repeated-measures analysis of variance (ANOVA) (IBM SPSS v.20.0).

**Results:**

The mean composite score was 3.18 of 4.0 (SD = 0.58; 95% CI [2.95–3.41]; range: 1.50–3.77), with 15 of 20 groups (75%) achieving good-to-excellent ratings (≥3.0). Excellent inter-rater reliability was confirmed: ICC (average) = 0.909 (95% CI [0.798–0.962]). Minor rater effects (*F* = 5.027, *p* = 0.012, η^2^ = 0.21) were overshadowed by substantial between-infographic variance (mean square [MS] ratio: 12.9:1).

**Conclusion:**

Scaffolded infographic assignments produced reliable Bloom’s “create”-level group products (ICC = 0.909) suitable for medical education assessment. The rubric demonstrates discriminative validity in assessing collaborative visual synthesis.

## Introduction

Technology integration has transformed undergraduate medical education, blending face-to-face and online environments to enhance teaching and learning (1). While online platforms offer flexibility and individualized access, challenges such as reduced engagement, screen fatigue, and competing priorities persist (2). Active visual tools, such as student-created infographics, address these challenges by promoting higher-order synthesis at Bloom’s “create” level, fostering collaboration and deep understanding in sustainable healthcare curricula (3, 4). Infographics, defined as visual representations that combine graphics, images, and minimal text to convey data, ideas, or processes, have gained traction as pedagogical tools in undergraduate medical education (5). Student-created infographics promote an active synthesis of complex topics such as sustainable healthcare, aligning with Bloom’s “create” level by requiring novel visual reorganization while reducing viewer cognitive load through hierarchical design (6).

Successful student-created medical infographics demand audience awareness, clear objectives, and iterative design (including color and font hierarchy and visual hierarchy), aligning with Bloom’s “create” level through drafting, peer-testing, and revision. This process equips learners with visual communication competencies essential for sustainable healthcare curricula (5, 6). This innovation is grounded in Bloom’s Revised Taxonomy, specifically targeting the create level—the highest level of cognitive processing, in which learners generate novel artifacts by reorganizing elements into new patterns or structures (4, 5). In this study, third-year medical students designed infographics visualizing sustainable healthcare topics (e.g., waste reduction and resource efficiency in healthcare systems), integrating data synthesis, evaluation, and visual reorganization. This approach fostered essential competencies in visual literacy, patient communication, and interdisciplinary thinking aligned with undergraduate medical education standards, distinguishing it from lower-level tasks such as remembering or analyzing (3).

Despite global adoption, infographic integration remains limited at the Alexandria University Faculty of Medicine, Egypt, particularly in online electives such as “Planetary Health and Sustainable Healthcare” (7). This study evaluated 20 student-created infographics after design training, scored by independent raters using a four-point rubric (content accuracy, design principles, visualization effectiveness, and communication clarity). The study addresses the following research question: What is the quality of infographics produced by third-year medical students in the Planetary Health and Sustainable Healthcare elective, as determined by expert ratings on a standardized four-point rubric? The specific research objectives are as follows:

1. To determine the mean, standard deviation, and distribution of scores assigned by expert raters to the infographics using the standardized four-point rubric.
2. To measure the inter-rater reliability of the expert assessments using a two-way mixed-effects ICC and repeated-measures ANOVA.

## Methods

### Study setting

This descriptive evaluation was conducted at the Faculty of Medicine, Alexandria University, Egypt, during 2022–2024. Approximately 206 third-year undergraduate medical students (enrollment: voluntary elective) participated in a 2-week fully online “Planetary Health and Sustainable Healthcare” course. We used Microsoft Teams for synchronous sessions and infographic training for both students and raters. In addition, we used Moodle (university learning management system [LMS], version 4.1) for asynchronous learning, assignment submission, and communication with students. Faculty included two medical educationists and two teaching assistants. Full course details, including quasi-experimental efficacy evaluation, are reported by Elhoshy et al. (7). The pedagogical format comprised (1) a 2-h synchronous infographic design training workshop (covering Bloom’s “create”-level principles and rubric criteria); (2) independent group student assignment for the creation of one infographic on assigned sustainable healthcare topics (e.g., healthcare waste reduction and resource-efficient practices); and (3) blinded evaluation by three independent raters (faculty with visual design/medical education expertise). The learning objective was for students to demonstrate Bloom’s “create”-level synthesis by producing high-quality infographics assessed through a standardized four-point rubric.

### Study design

This descriptive evaluation systematically assessed the quality and reliability of all 20 group-created infographics (group products, not individual student performance) as Bloom’s “create”-level artifacts in undergraduate medical education. The design followed a structured five-step process:

Step 1: participant recruitment and cohort formation

The elective course enrolled 20–30 students per run. Students were divided into small groups of no more than 10 students each. The course was repeated across semesters, yielding a total of 206 students. For analysis, we selected 20 student groups that produced 20 infographics (a purposive subsample for depth; no exclusions).

Step 2: synchronous training workshop

Participants attended a mandatory 2-h Microsoft Teams infographic design training led by course instructors (two medical educationists), covering Bloom’s “create”-level principles (evidence synthesis into novel visuals), revised rubric criteria (detailed below), and Piktochart tools. Training included scored practice examples, common pitfalls, and rater calibration.

Step 3: group assignment and topic allocation

Instructors randomly assigned students to 20 collaborative groups (no more than 10 students per group). Planetary healthcare topics were equitably distributed: (1) Climate-resilient healthcare systems, (2) Greenhouse gas emissions’ healthcare effects, (3) Water crisis as health crisis, (4) Heat waves/global temperatures, (5) Climate change impacts on children’s health, (6) Climate change impacts on women’s health, (7) Climate change impacts on human nutrition, and (8) Green healthcare systems.

Step 4: infographic creation period

Third-year medical students’ groups were given 1 week for independent infographic creation, progressing through research, storyboard drafting, design development, internal peer review, and final submission. Students submitted both a Microsoft Word file (containing full research details: 8–12 core information units from 3 to 5 peer-reviewed sources, comprehensive evidence, and references) and one streamlined infographic per group (20 total) through Moodle, with infographics focusing exclusively on content synthesis and data visualization (image-to-text ratio 70:30, hierarchical structure).

**Figure 1:**
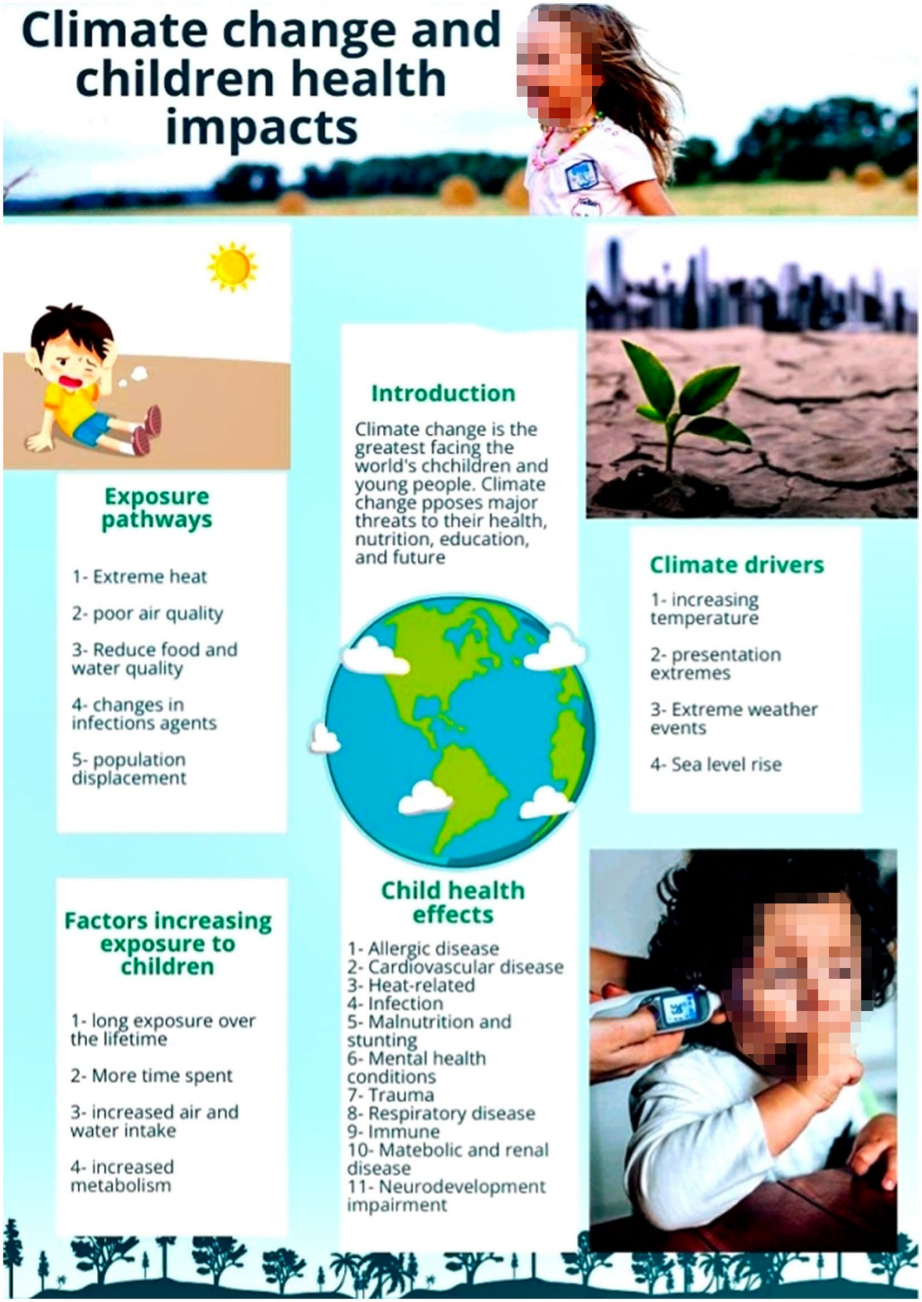
An infographic created by medical students in a sustainable healthcare course, illustrating the pathways between climate drivers and pediatric health outcomes.

Figure 1 shows an example of an infographic developed by students titled “The impact of climate change on human nutrition.” This representative example demonstrates typical Bloom’s “create”-level synthesis and hierarchical structure (title, key message, and data visualizations).

Step 5: blinded expert evaluation

Three independent raters (medical educationists with experience in visual design; selection criteria: ≥5 years’ experience and prior infographic assessment) underwent 1-h training (practice scoring and disagreement resolution through discussion). Raters, blinded to groups/course context, scored all 20 infographics using a revised rubric [Table 1; adapted from Texas Education Agency (8)]. We adapted this infographic rubric as it emphasizes content accuracy (50% weight) over esthetics, matching Bloom’s “create” synthesis requirements; in addition, it uses a four-point scale with a clear explanation of each score, which optimizes discrimination for expert raters; finally, it was pilot-tested with three infographics during rater training, yielding a pre-study ICC of 0.82. This rubric balances content rigor with visual communication, distinguishing it from design-heavy alternatives. Each of the three blinded raters scored every infographic across 12 criteria grouped into four domains (content 50%, focus 20%, visual appeal 20%, and mechanics 10%; see Table 1).

1. Raw scores: 0–4 per criterion (4 = exceptional).
2. Domain composites: Weighted average within each domain, normalized to 0–4.0 scale (e.g., content = [sum weighted criteria]/max possible).
3. Infographic score: Average of rater’s four domain composites (overall 0–4.0): ${\overset{ˉ}{Q}}_{i}=\frac{Q_{i,1}+Q_{i,2}+Q_{i,3}}{3}$ (*Q_{i,r}* = rater *r*’s composite for infographic *i*).

**Table 1:** Infographic evaluation rubric.

Criterion	Weight	Description	Exceptional (4)	Admirable (3)	Marginal (2)	Unacceptable (1)
Content	50%	≥8 synthesized units from Word file, ≥3 citations, hierarchical structure, and ≥2 quantitative visualizations	Comprehensive, error-free synthesis	Mostly accurate	Some inaccuracies	Factually wrong
Focus	20%	Clear main message	Crystal clear thesis	Clear message	Somewhat unclear	No focus
Visual appeal	20%	Professional hierarchy	Professional design	Attractive	Basic design	Poor esthetics
Mechanics	10%	Flawless execution	Flawless	Minor errors	Noticeable errors	Major errors

### Statistical methods

Descriptive statistics included mean scores (±SD) for each rubric criterion (content, focus, visual appeal, and mechanics) and overall composite scores (maximum 4 points) across 20 infographics. Inter-rater reliability was quantified using the two-way random-effects intraclass correlation coefficient (ICC) with 95% confidence intervals for absolute agreement (single and average measures). A one-way ANOVA with *post-hoc* Tukey’s honestly significant difference (HSD) tested differences between raters’ mean scores, with statistical significance set at a *p*-value of <0.05. Analyses were conducted using IBM SPSS Statistics, version 20.0 (IBM Corp., Armonk, NY, USA).

## Results

### Infographic quality (group-level)

Blinded evaluation of 20 collaborative group infographics (≤10 students per group) yielded a mean composite quality score of 3.18 out of 4.0 (SD = 0.58; 95% CI [2.95–3.41]; range: 1.50–3.77), demonstrating group proficiency at Bloom’s “create” level for synthesizing sustainable health concepts into professional visuals. Notably, 15 of 20 (75%) infographics achieved good-to-excellent ratings (≥3.0/4.0), and 6 of 20 (30%) infographics achieved high excellence (≥3.5/4.0) (see Table 2).

**Table 2:** Rater scores and infographic means (*n* = 20).

Infographic group ID	Rater 1	Rater 2	Rater 3	Mean	(SD)
1	3.7	3.2	3.2	3.40	(0.26)
2	3.6	3.6	3.7	3.63	(0.06)
3	3.4	3.1	3.4	3.30	(0.17)
4	3.5	3.0	3.5	3.33	(0.29)
5	3.4	3.0	3.4	3.27	(0.24)
6	3.6	3.3	3.7	3.53	(0.21)
7	3.3	3.2	3.2	3.23	(0.06)
8	2.5	2.4	4.0	2.97	(0.89)
9	3.3	3.3	3.7	3.43	(0.23)
10	3.2	3.1	3.5	3.27	(0.21)
11	4.0	3.2	3.7	3.63	(0.40)
12	3.7	3.7	3.9	3.77	(0.10)
13	3.6	3.6	3.7	3.63	(0.06)
14	3.6	3.5	3.7	3.60	(0.10)
15	2.7	2.7	2.5	2.63	(0.11)
16	2.1	2.1	2.3	2.17	(0.11)
17	1.0	1.8	1.7	1.50	(0.39)
18	3.1	3.2	3.0	3.10	(0.10)
19	2.8	2.8	3.2	2.93	(0.23)
20	3.5	3.3	3.4	3.40	(0.10)
Rater M (SD)	3.18	3.06	3.32		
Rater SD	(0.69)	(0.49)	(0.57)		
Overall				3.18	(0.58)

### Inter-rater reliability

The two-way mixed-effects ICC model is appropriate for our design in which three fixed expert raters evaluate 20 group infographics. The model quantified both absolute agreement and consistency (Table 3). The results confirmed outstanding inter-rater reliability: average-measures ICC = 0.909 (95% CI [0.798–0.962])—exceeding the >0.90 threshold for high-stakes educational assessment, and single-measures ICC = 0.768 (95% CI [0.568–0.893])—robust even using one rater. Both are highly significant (*F* = 12.929, *p* < 0.001), indicating that the rubric produces stable group quality judgments suitable for Bloom’s “create”-level evaluation.

**Table 3:** Intraclass correlation coefficients (two-way mixed-effects model).

Measure	ICC	95% CI	F	df1	df2	*p*
Single measures	0.768	0.568–0.893	12.929	19	38	<0.001
Average measures	0.909	0.798–0.962	12.929	19	38	<0.001

**Figure 2:**
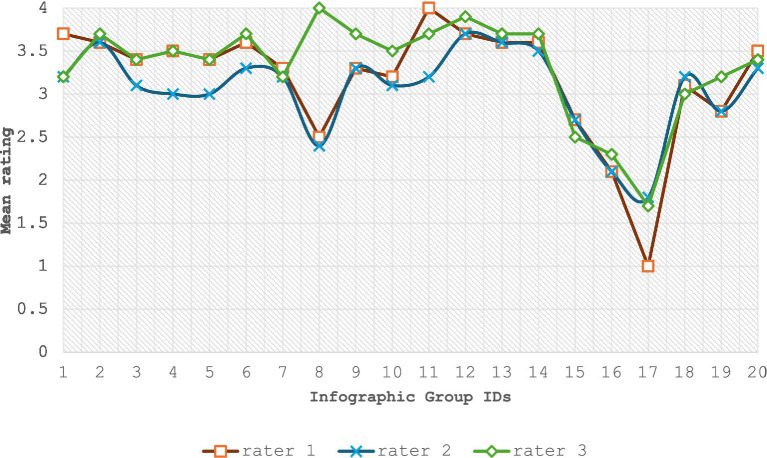
Comparison of rating patterns across 20 infographics by three independent raters.

Supplementary Pearson’s correlations (Table 4) further demonstrated strong rater association in ranking infographics. The pairwise correlations were ranked as follows: Raters 1–2 (*r* = 0.910) had the strongest correlation, followed by Raters 1–3 (*r* = 0.798) and Raters 2–3 (*r* = 0.751)—all exceeding 0.70. Figure 2 visualizes these patterns: raters showed the highest consensus on the three lowest-quality infographics (15–17; all scored <2.6/4.0) and progressive convergence for the final infographics (18–20). Greatest variability occurred mid-range (infographics 8–11), where fine quality distinctions proved challenging, as expected in rubric validation.

**Table 4:** Inter-rater correlation matrix for three independent raters.

Rater	Rater 1	Rater 2	Rater 3
Rater 1	1.000	0.910	0.798
Rater 2	0.910	1.000	0.751
Rater 3	0.798	0.751	1.000

Together, ICC primacy with correlational support establishes this rubric’s scalability for medical education programs evaluating collaborative visual synthesis.

### Rater differences

Repeated-measures ANOVA detected statistically significant but practically small rater differences (*F* = 5.027, *p* = 0.012, η^2^ = 0.21; Table 5)—explaining only 21% of the variance. Tukey’s HSD identified Rater 3 as systematically higher than Rater 2 (*p* = 0.011); no other pairwise differences were observed. Critically, the between-infographic mean square (MS = 0.904) significantly exceeded the residual error (MS = 0.070)—a ratio of 12.9:1—demonstrating that the rubric primarily captures the true quality differences between infographics rather than rater subjectivity. This supports the rubric’s discriminative validity for distinguishing group performance levels.

**Table 5:** A one-way repeated measures ANOVA comparing rater differences.

Source	Sum of squares	df	Mean square	F	*p*
Infographics	17.176	19	0.904		
Raters	0.703	2	0.352	5.027	0.012
Residual	2.657	38	0.070		

## Discussion

This descriptive evaluation demonstrated that a mean composite score of 3.18 out of 4.0 (SD = 0.58), with 15 of 20 groups (75%) achieving good-to-excellent ratings, indicates group-level synthesis capability consistent with Bloom’s “create” expectations for collaborative visual products. Outstanding reliability (ICC average = 0.909, 95% CI [0.798–0.962]) confirms the rubric’s precision for distinguishing quality despite minor rater effects (*p* = 0.012, η^2^ = 0.21). Groups excelled in content synthesis (50% weight) while showing expected variability in visual execution, aligning with novice designer trajectories. High-quality outputs indicate synthesis capability but require cognitive process evidence (e.g., design logs) for full Bloom’s validation.

Infographic assignments offer a structured, practical addition to online medical electives through a five-step process: a 2-h medical student training workshop covering infographic design principles and rubric criteria; group formation of no more than 10 students per infographic to reduce individual workload; a 1-week creation window with topic templates; and blinded three-rater scoring that confirmed excellent reliability (ICC = 0.909). This process yielded 75% good-to-excellent outputs from first-time creators, demonstrating feasibility for planetary and sustainable healthcare curricula. Faculty development is minimal, requiring only a 1-h rater training session focused on explicit rubric domains. The approach is cost-effective, leveraging free tools such as Piktochart and a group model that produced 20 infographics. Assessment efficiency is enhanced through composite scoring for rapid grading, while sustainable healthcare topics bridge clinical and environmental competencies (5, 9, 10).

This study’s mean quality score of 3.18 out of 4.0 aligns closely with the findings of Shanks et al. (5), who evaluated undergraduate public health infographics and reported that students achieved comparable high-quality visual outputs following similar training protocols. However, unlike the study by Shanks et al., which focused on basic visualization competencies, our application at Bloom’s “create” level required the novel synthesis of sustainable health concepts, with top performers (infographic 12: 3.77) demonstrating professional-grade integration of clinical and environmental data. Quality variation (1.50–3.77) mirrors the findings of Jahan et al. (11) on health education infographics, where design clarity challenges affected 25%–30% of submissions despite strong content. Our rubric’s content weighting (50%) mitigated this issue by prioritizing medical accuracy over esthetics—unlike Jahan’s equal-weighted approach—resulting in 75% good-to-excellent ratings vs. their 65%, confirming that weighted criteria enhance discrimination at Bloom’s “create”-level synthesis.

Average-measures ICC of 0.909 (95% CI: 0.798–0.962) demonstrates excellent inter-rater reliability (12). This value exceeds the single-rater ICC of 0.82 for first-year medical student visualizations found by Akers et al. and the pairwise r of 0.78 for biochemistry bioinfographics found by McCulloch et al., despite our third-year cohort addressing higher-order Bloom’s “create”-level synthesis rather than basic data presentation (9, 10). Single-measures ICC of 0.768 confirms individual rater consistency, while narrow CIs and *F*(19,38) = 12.929 (*p* < 0.001) validate the two-way mixed-effects model. Although the ANOVA detected minor systematic rater differences (*F* = 5.027, *p* = 0.012), the between-infographic mean square variance (0.904) dwarfed the residual error (0.070; ratio 12.9:1), indicating rubric sensitivity to true quality differences rather than evaluator subjectivity (12, 13).

Despite the demonstrated benefits of infographics in fostering Bloom’s “create”-level synthesis among medical students, implementation challenges persist, including heterogeneous prior design experience that influenced output quality (range: 1.50–3.77) and initial resource gaps addressed through targeted workshops. Previous literature emphasizes mandatory scaffolded training and multi-rater validation (ICC = 0.909) to mitigate subjectivity, aligning with best practices for visual health communication (14, 15). This study underscores infographics’ potential as a practical curricular tool when paired with structured support, equipping future physicians for patient-centered visual literacy while highlighting the need for multi-institutional trials and longitudinal skill assessment to confirm sustained impact.

### Study limitations

This descriptive evaluation carries inherent methodological constraints, capturing a single implementation snapshot that precludes causality assessment or longitudinal tracking of skill development. The modest sample of 20 group infographics from voluntary elective participants at Alexandria University introduces selection bias and limits generalizability to diverse medical curricula or global contexts. Context-specific development within one course may not reflect infographic efficacy across disciplines, while group outputs obscure individual contributions. Although three-rater inter-rater reliability proved robust (ICC = 0.909), residual rater subjectivity persists despite standardized rubrics.

### Study delimitations

This evaluation intentionally focused on a single elective implementation at Alexandria University to establish proof-of-concept for infographic integration in undergraduate medical education. The scope was delimited to 20 group-produced infographics on sustainable health topics, excluding individual assessments or other visual formats (e.g., posters and videos). Rubric application was confined to three expert raters with medical education expertise, prioritizing feasibility over exhaustive validation panels. No student perception data, learning outcomes, or comparative controls were included, as the primary aim was descriptive quality assessment rather than causal inference or efficacy testing. These boundaries ensure targeted, replicable insights while acknowledging opportunities for broader future inquiry.

### Future directions

Future research should prioritize longitudinal studies tracking infographic creators’ visual literacy into clinical rotations, measuring sustained impacts on patient communication and health promotion. Multi-institutional collaborations across more medical schools could validate scalability, targeting more infographics with diverse demographics and curricula. Randomized controlled trials comparing infographics to traditional assignments (reports and posters) will quantify pedagogical gains in synthesis and retention. Integrating AI design assistants and student co-design workshops could address skill gaps, while validated perception surveys assess self-efficacy and transferability to real-world health campaigns. Exploration of digital platforms for interactive infographics and global health topics promises enhanced engagement and prepares physicians for data visualization in evidence-based practice.

## Conclusion

This descriptive evaluation confirms infographics as a feasible Bloom’s “create”-level assessment tool in undergraduate medical education through scaffolded workshops and group collaboration at Alexandria University. Despite context-specific delimitations and implementation challenges such as heterogeneous design experience, the robust between-infographic variance demonstrates rubric sensitivity to true quality differences over rater subjectivity. Structured integration—emphasizing training, multi-rater validation, and content weighting—equips future physicians with visual literacy for patient-centered communication, supporting the need for multi-institutional trials and longitudinal assessment to optimize curricular scalability.

## Data Availability

The raw data supporting the conclusions of this article will be made available by the authors, without undue reservation.
